# Supplementary material for: Polygodial, a Sesquiterpene Dialdehyde, Activates Apoptotic Signaling in Castration-Resistant Prostate Cancer Cell Lines by Inducing Oxidative Stress
Source: Cancers (Basel). 2022 Oct 26;14(21):5260. doi: 10.3390/cancers14215260 (PMC9656647; doi:10.3390/cancers14215260)
Supplement: Supplementary file 1 [file cancers-14-05260-s001.zip › cancers-1950536-supplementary.pdf]

PC3-TXR

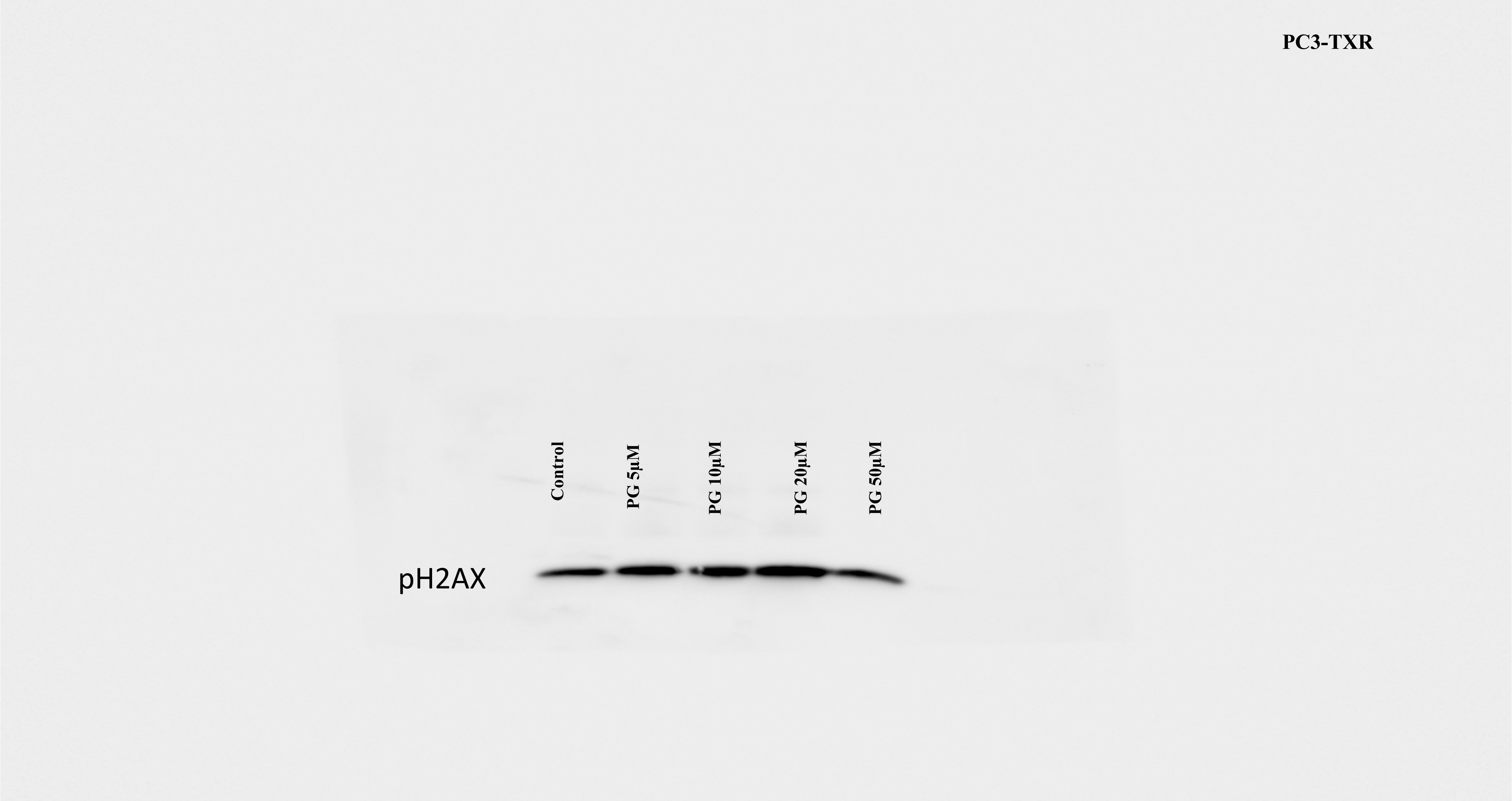
PC3-TXR
Control
PG 5µM
PG 10µM
PG 20µM
PG 50µM
pH2AX

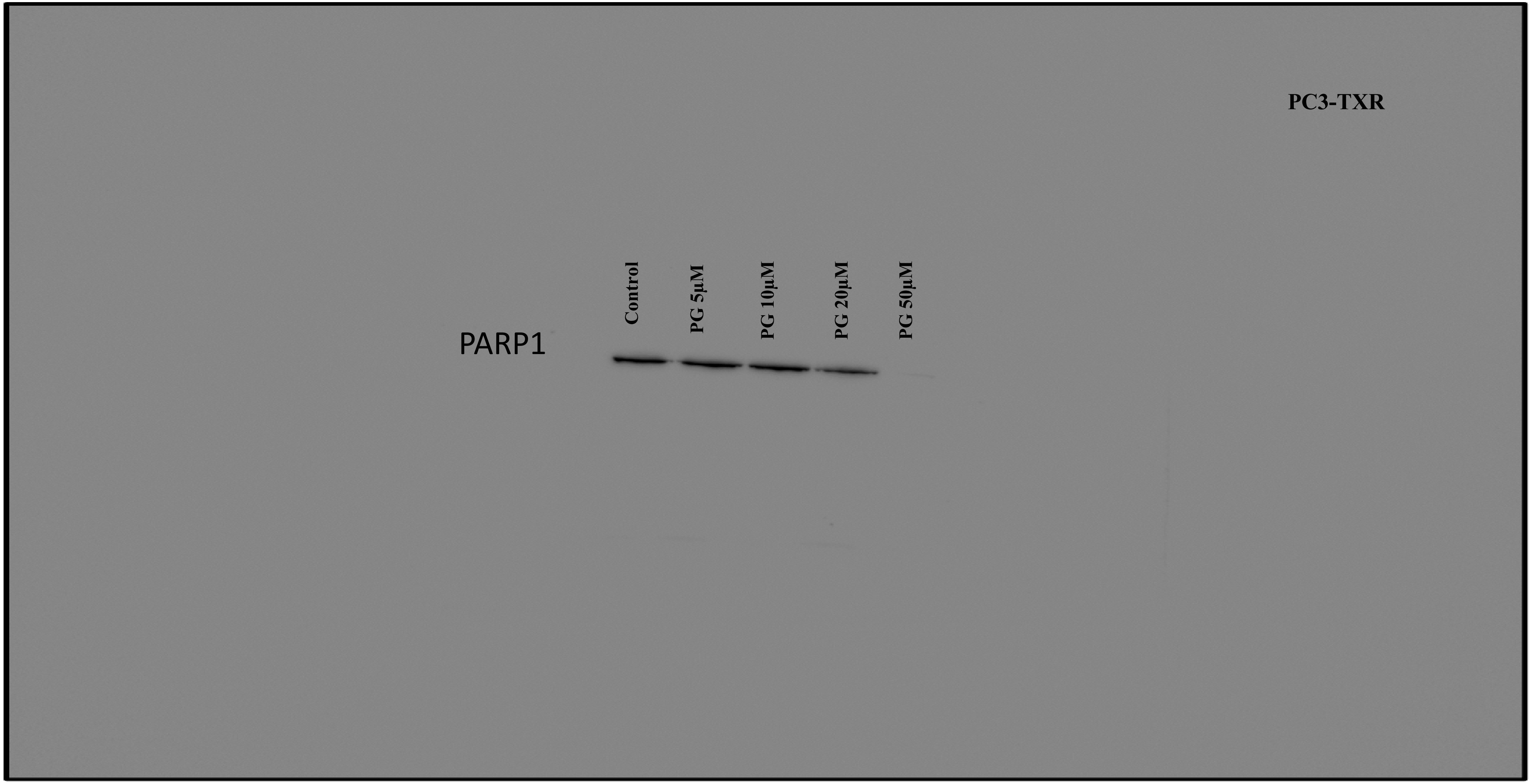
PC3-TXR
PARP1
Control
PG 5µM
PG 10µM
PG 20µM
PG 50µM

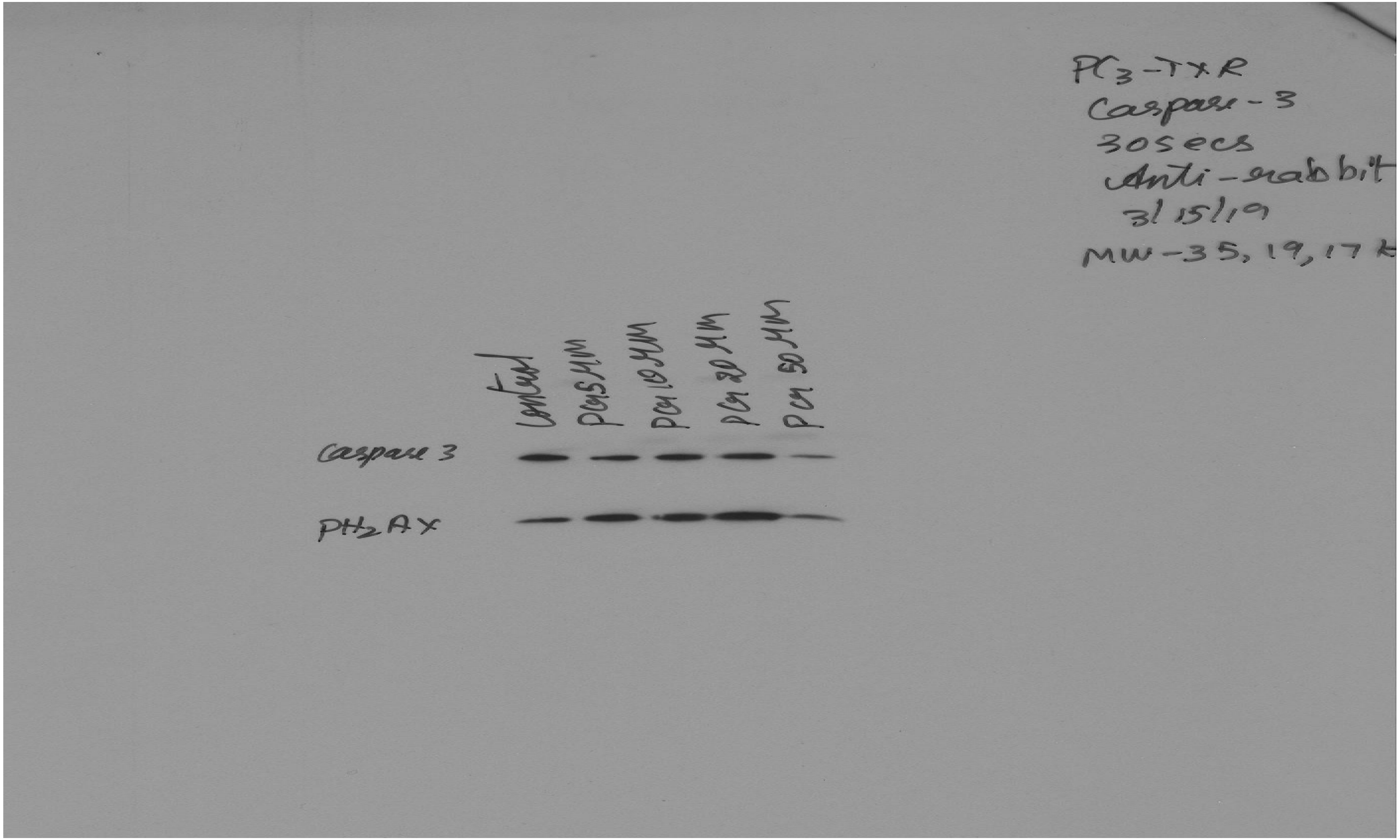
PC3-TXR
Caspase-3
30secs
Anti-rabbit
3/15/19
MW-35, 19, 17 k
Control
PCa 5 μM
PCa 10 μM
PCa 20 μM
PCa 50 μM
Caspase 3
PH2AX

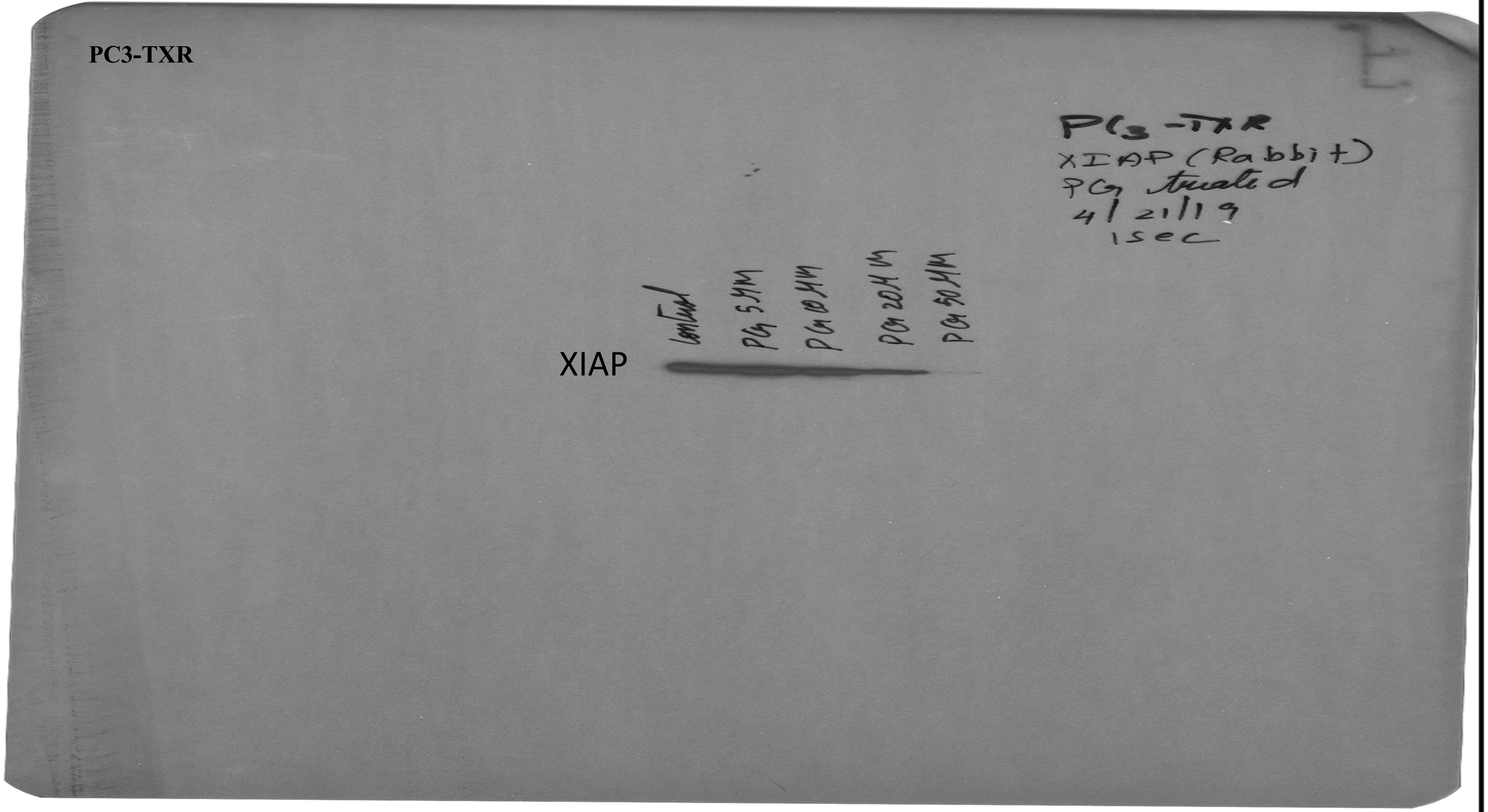
PC3-TXR
XIAP
PC3-TXR
XIAP (Rabbit)
PG treated
4/21/19
1sec
Control
PG 5 μM
PG 10 μM
PG 20 μM
PG 50 μM

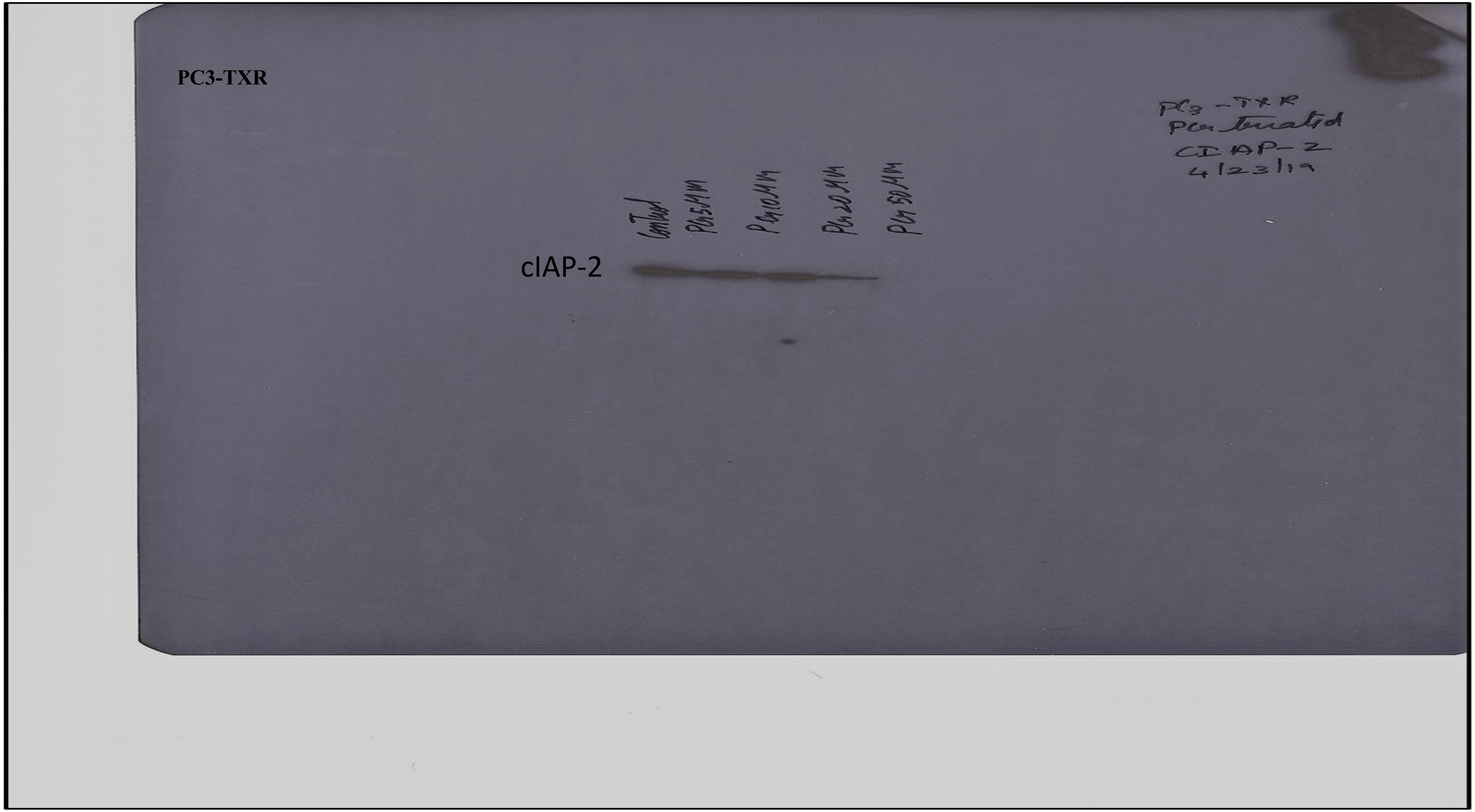
PC3-TXR
cIAP-2

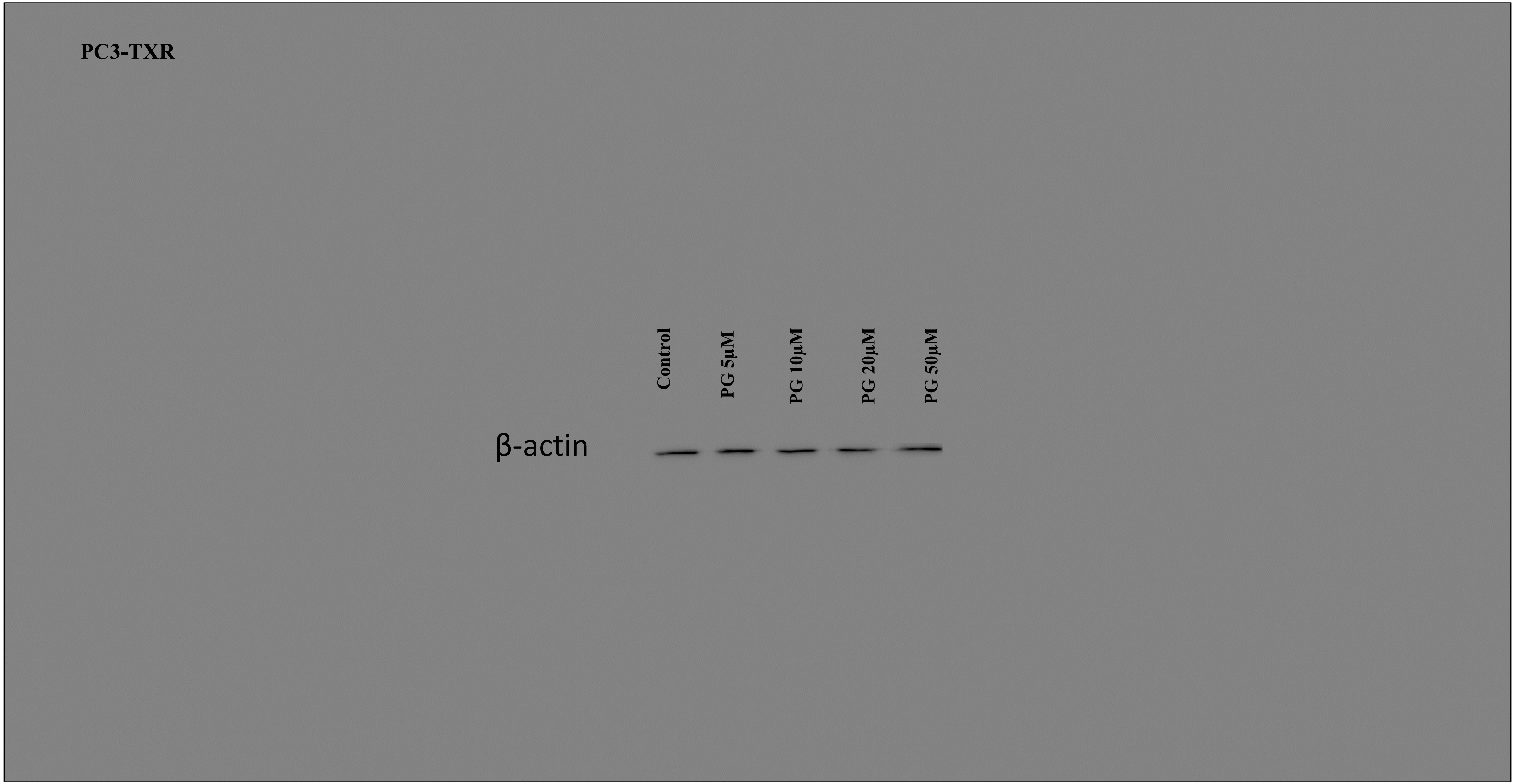
PC3-TXR
Control
PG 5μM
PG 10μM
PG 20μM
PG 50μM
β-actin

Cleaved PARP1

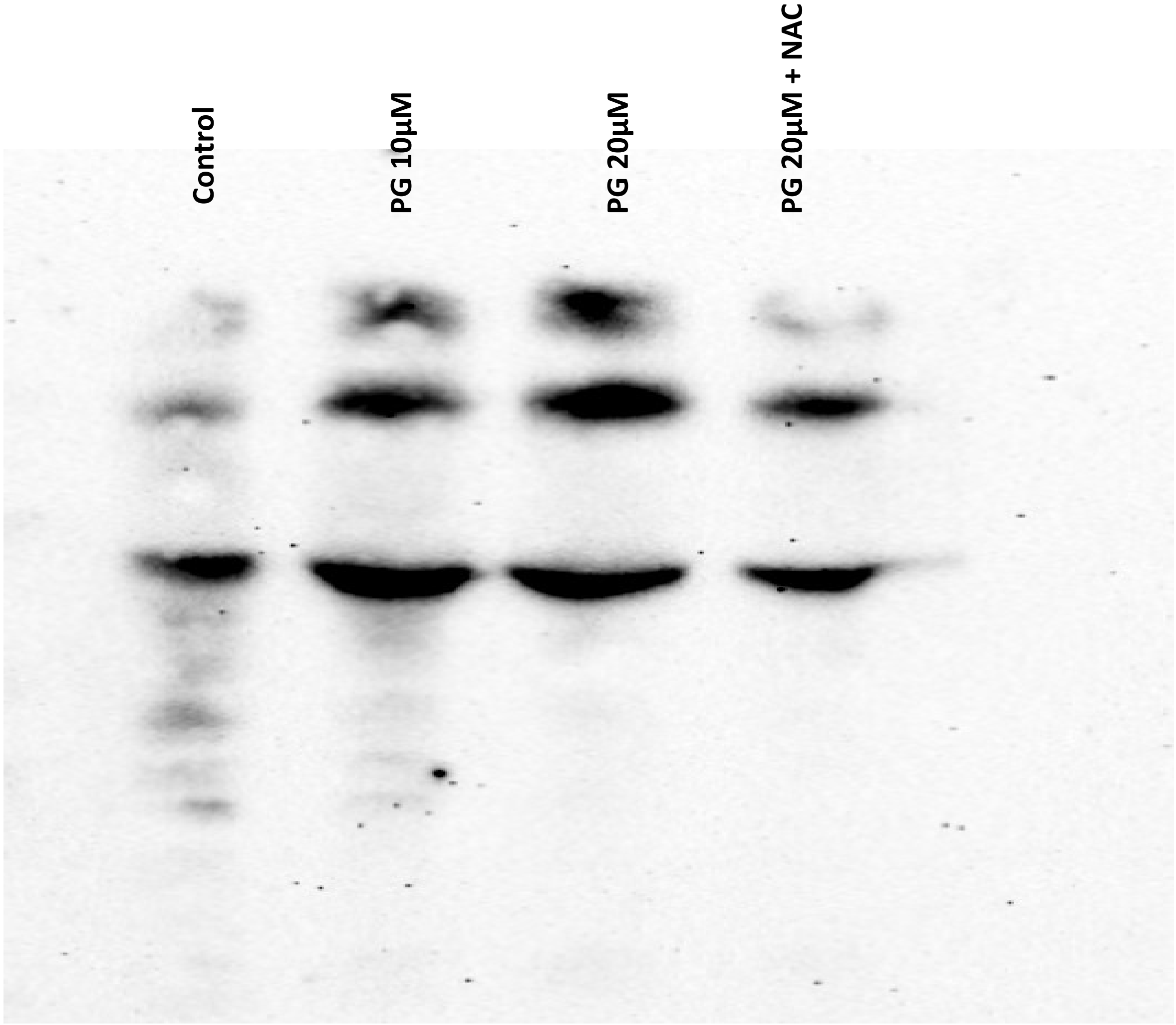

Beta- actin

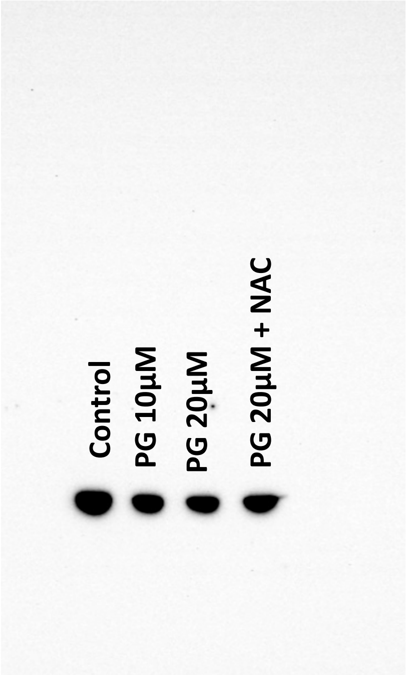

DU145-TXR

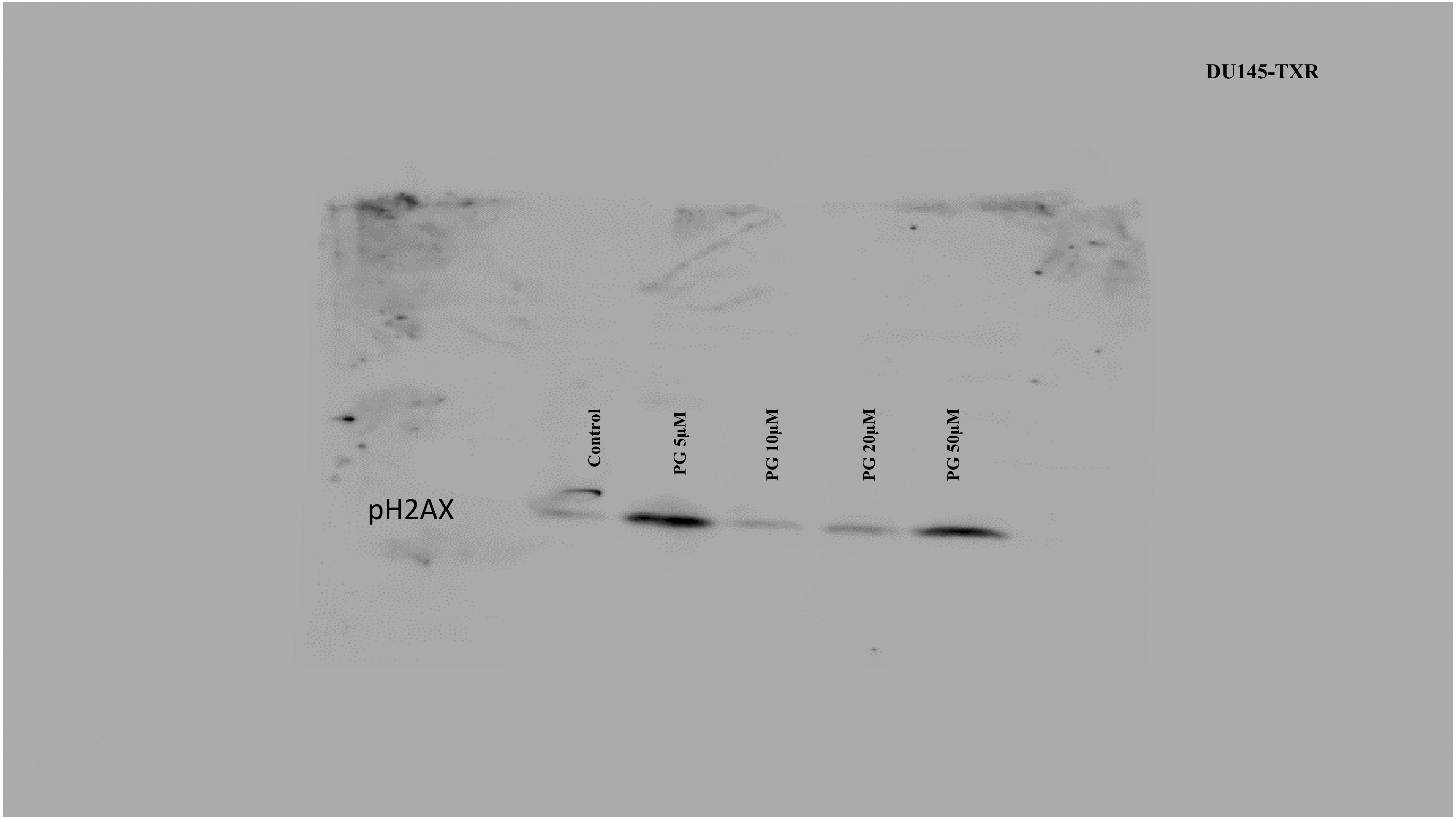
DU145-TXR
Control
PG 5µM
PG 10µM
PG 20µM
PG 50µM
pH2AX

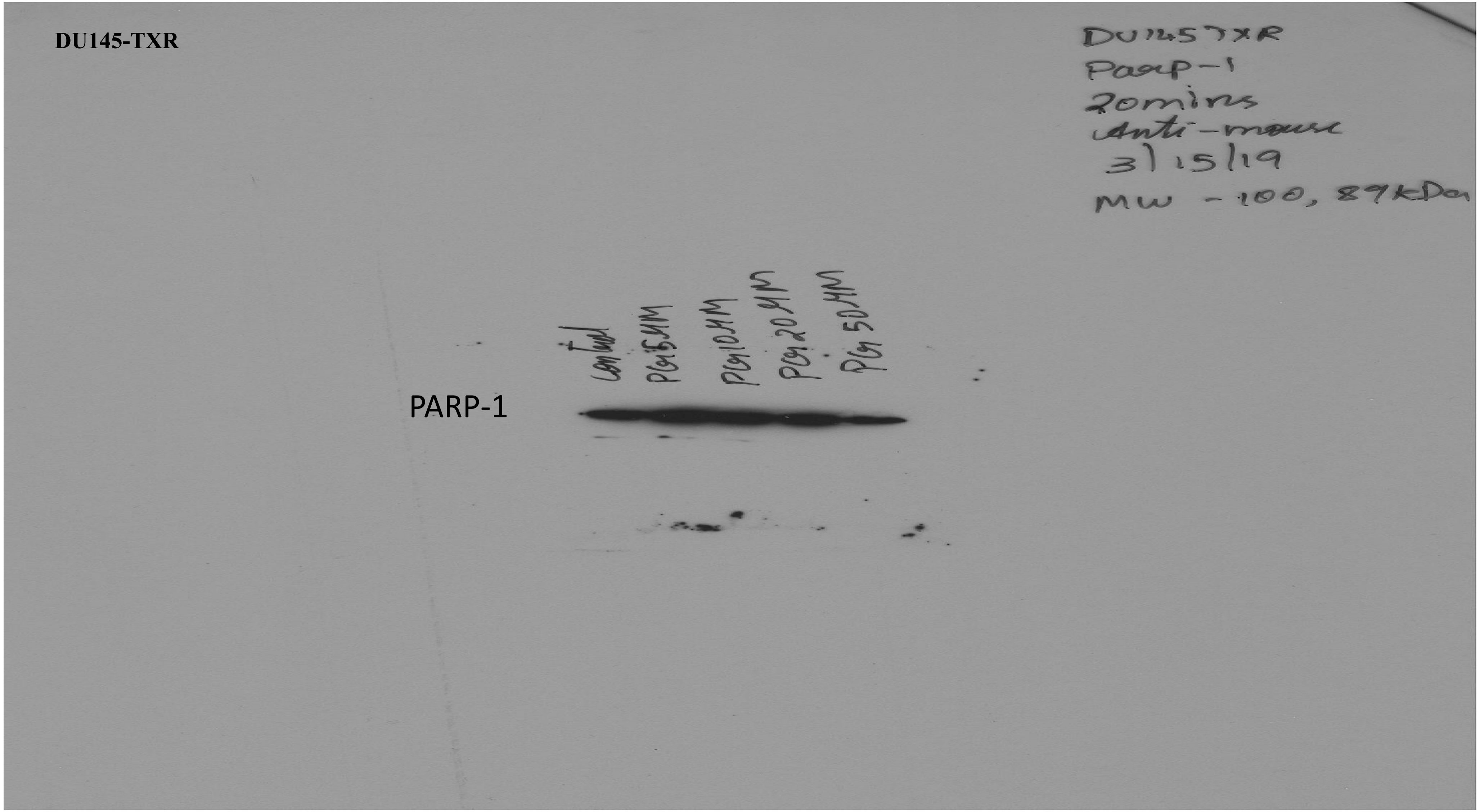
DU145-TXR
PARP-1
DU145TXR
Parp-1
20mins
Anti-mouse
3/15/19
MW -100, 89kDa
control

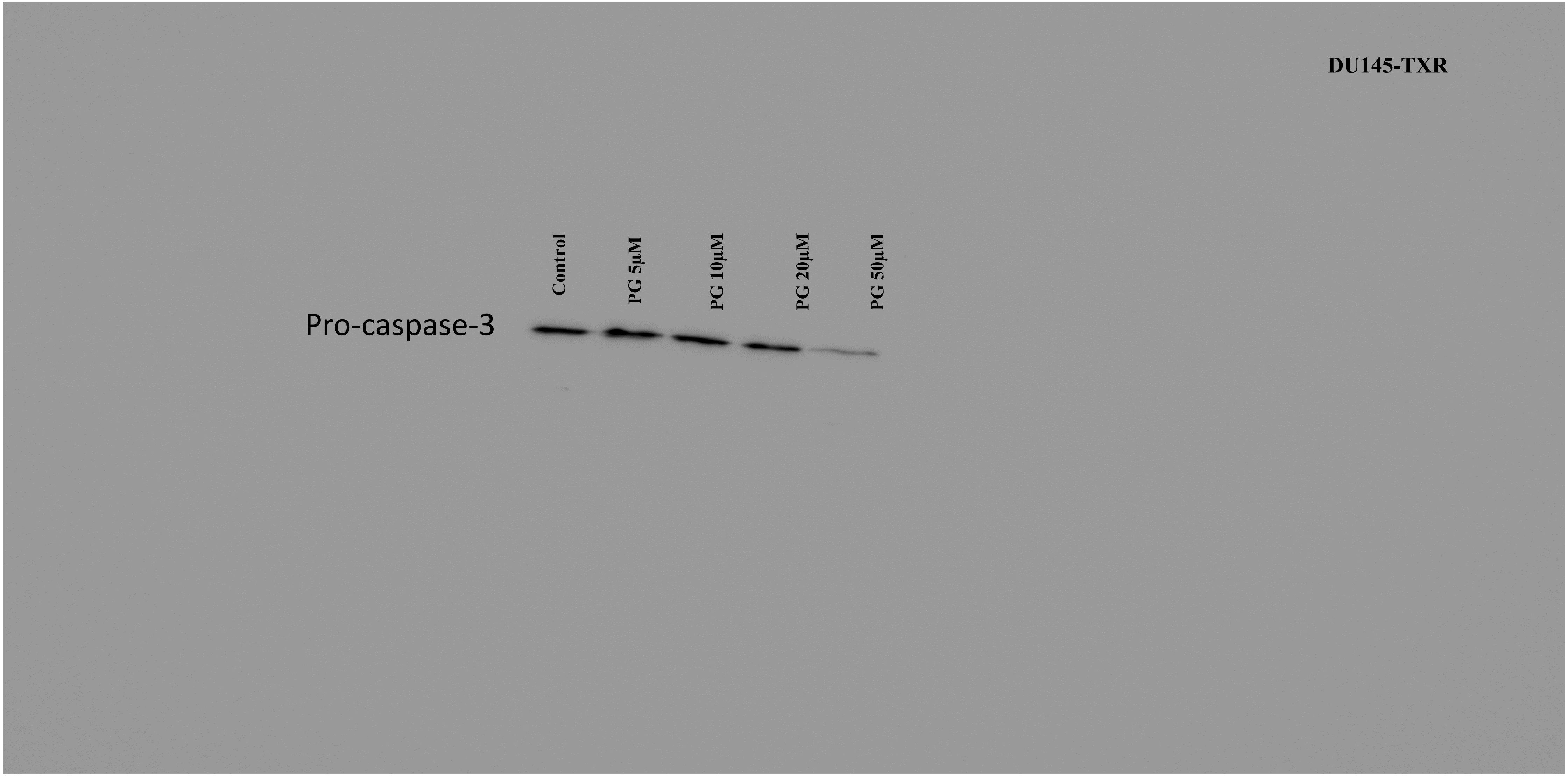
DU145-TXR
Control
PG 5μM
PG 10μM
PG 20μM
PG 50μM
Pro-caspase-3

DU145-TXR

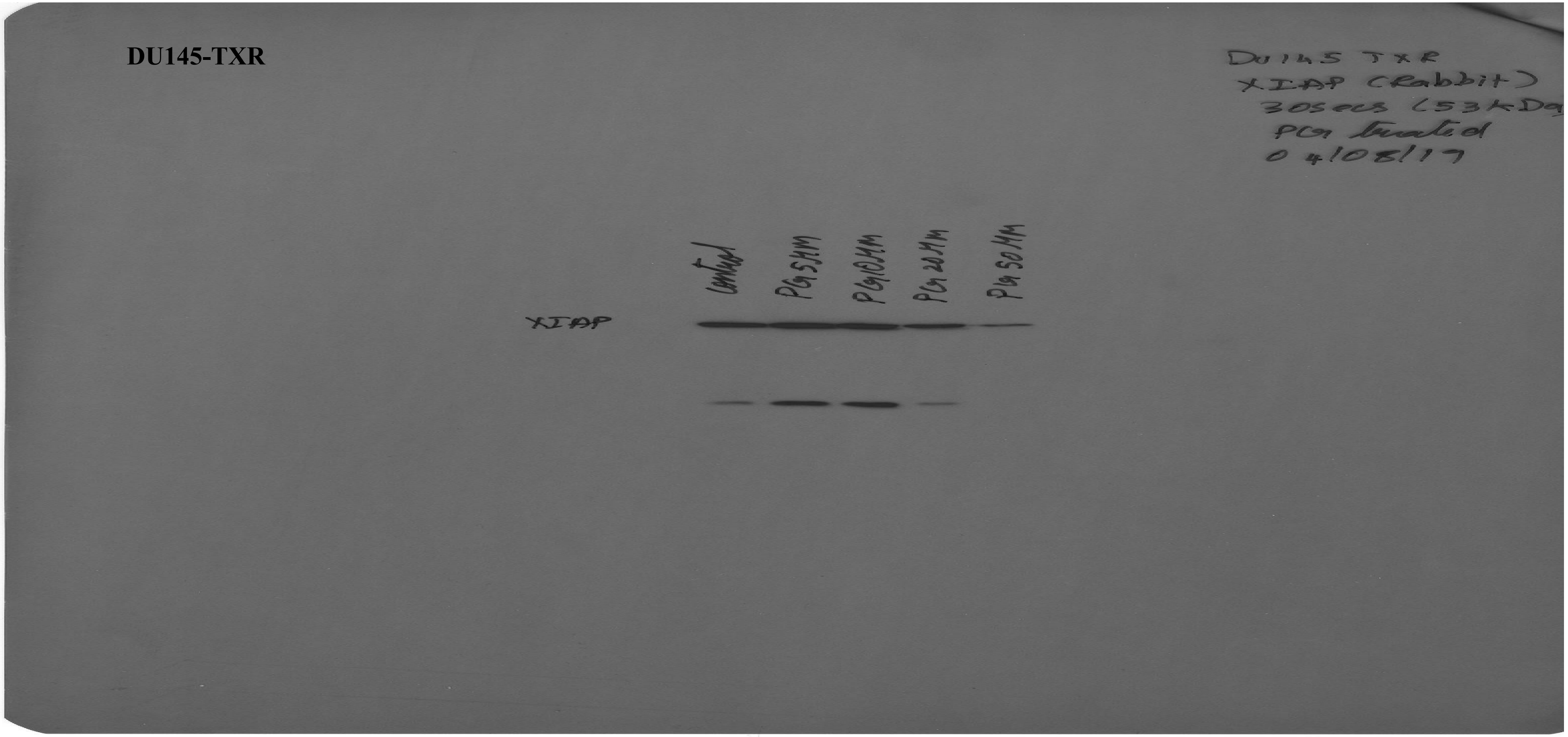

DU145 TXR
XIAP (Rabbit)
30secs (53kDa
PCa treated
04/08/17
control
PG 5μM
PG 10μM
PG 20μM
PG 50μM
XIAP

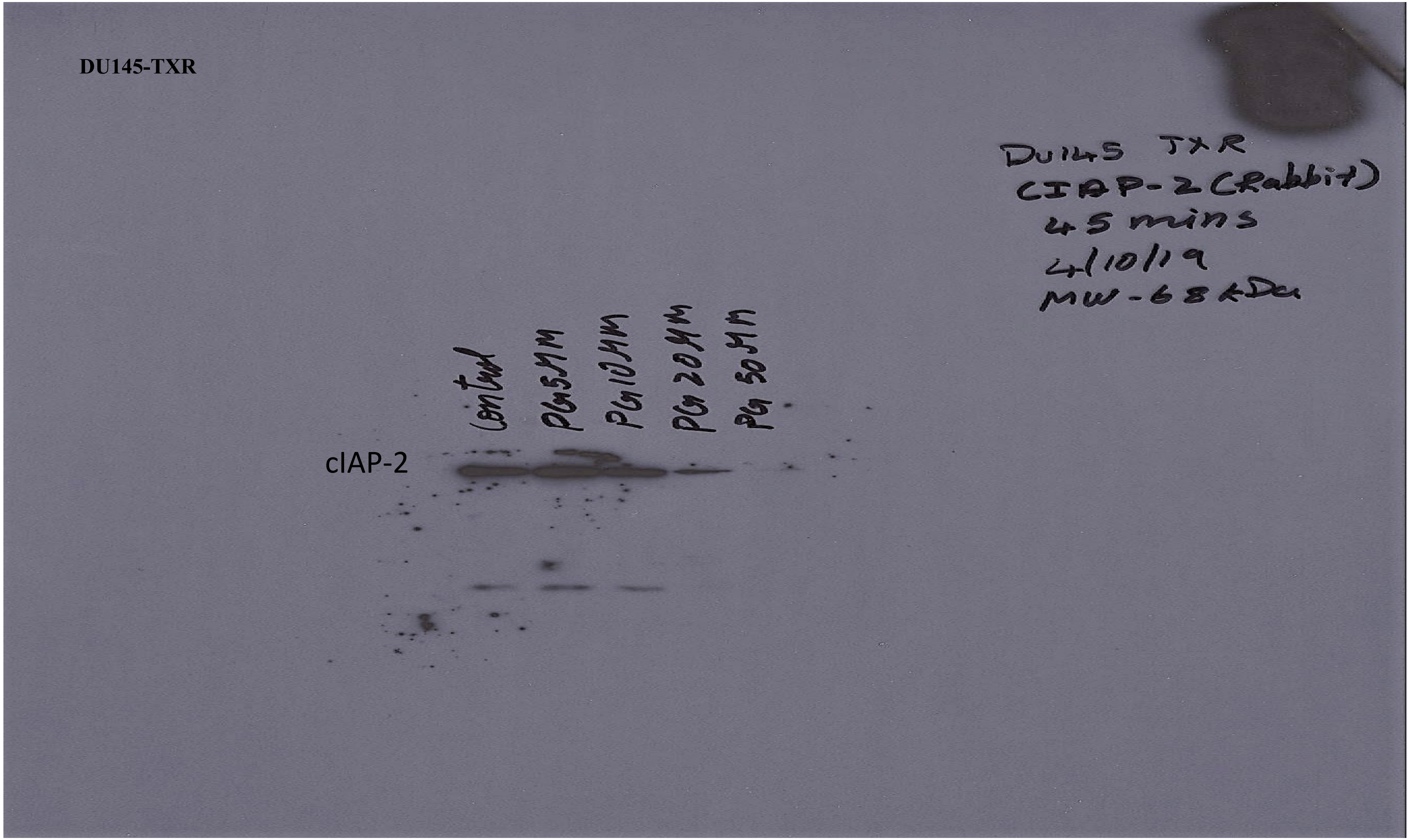
DU145-TXR
cIAP-2
DU145 TXR
CIAP-2 (Rabbit)
45 mins
4/10/19
MW-68 kDa
Control
PG 5 μM
PG 10 μM
PG 20 μM
PG 50 μM

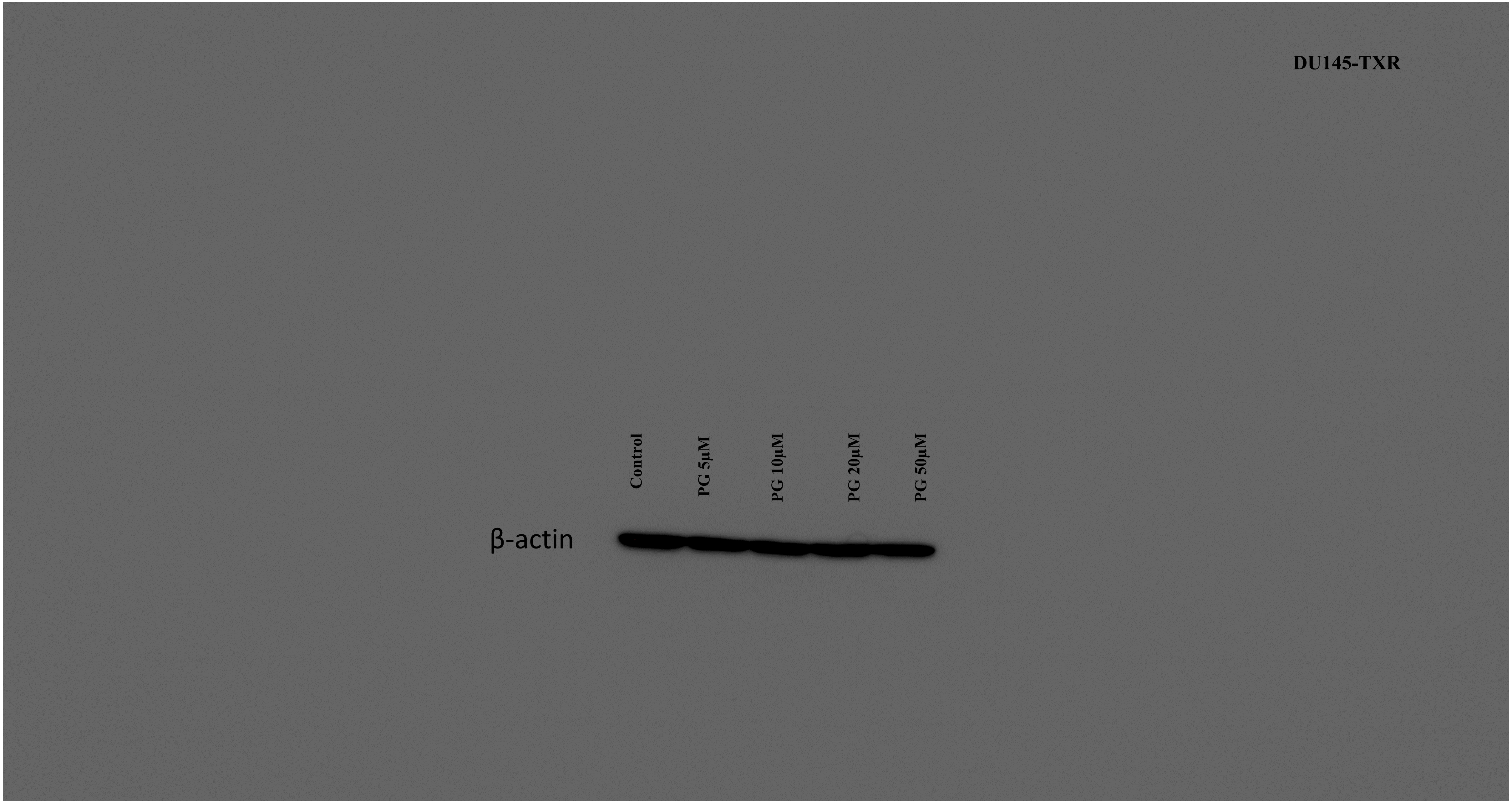
DU145-TXR
Control
PG 5μM
PG 10μM
PG 20μM
PG 50μM
β-actin

Cleaved PARP1

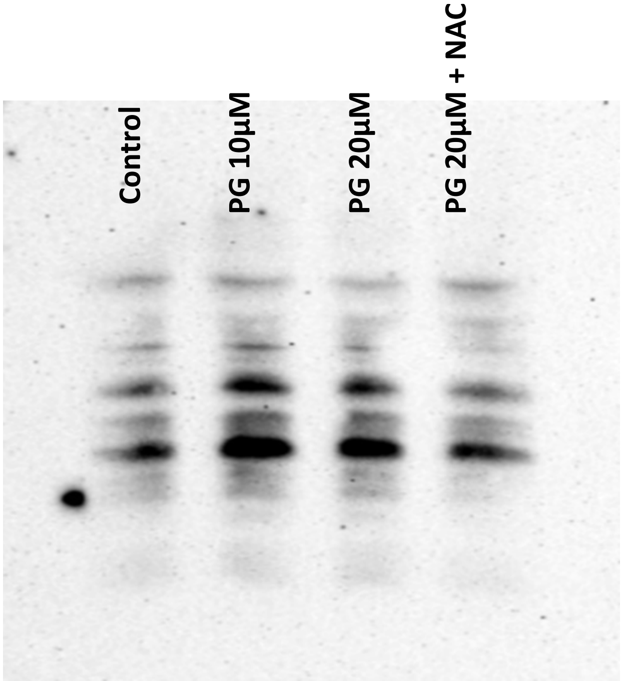

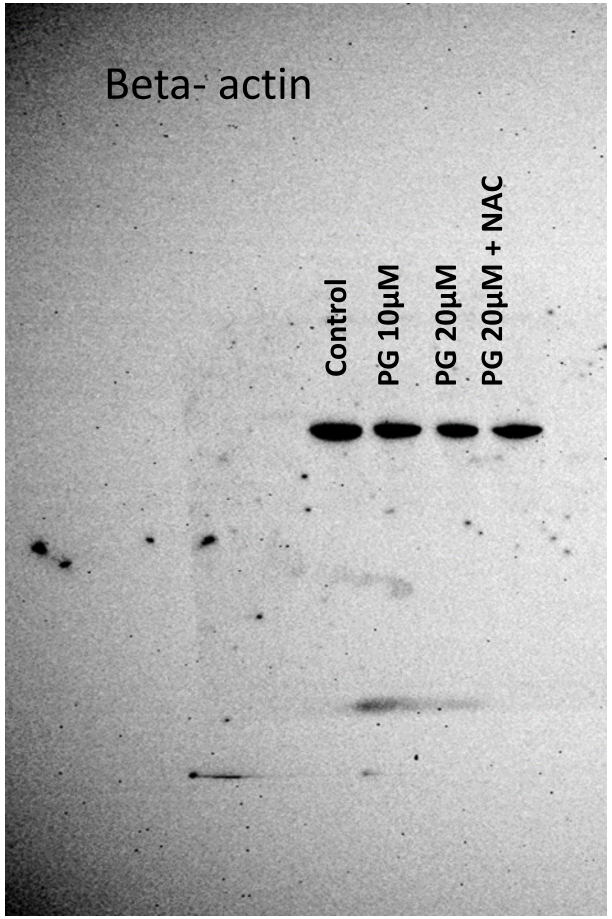
Beta- actin
Control
PG 10µM
PG 20µM
PG 20µM + NAC
